# Climate-Driven Shifts in Crimean-Congo Hemorrhagic Fever Epidemiology in Kazakhstan

**DOI:** 10.5334/aogh.5327

**Published:** 2026-07-24

**Authors:** Fariza Khozhamkul, Turliyev Zangar, Rabiga Khozhamkul, Aizhan Esmagambetova, Manar Smagul, Tuleuov Akan

**Affiliations:** 1 National Center of Public Health Care of the Ministry of Health of the Republic of Kazakhstan, Chief specialist of the Department for Work with International Organizations, Almaty, Kazakhstan; 2 Scientific and Practical Center for Sanitary and Epidemiological Expertise and Monitoring and the National Center for Public Health of the Ministry of Healthcare of the Republic of Kazakhstan, Head of the Department for the Prevention of Infectious Diseases, Almaty, Kazakhstan; 3 Asfendiyarov Kazakh National Medical University, teacher at Department of Biostatistics and Basic of Research, Almaty, Kazakhstan; 4 Al-Farabi Kazakh National University, teacher at the Department of health policy and public health, Almaty, Kazakhstan; 5 National Center of Public Health Care of the Ministry of Health of the Republic of Kazakhstan, Chief specialist of the Department for Work with International Organizations, Astana, Kazakhstan

**Keywords:** Crimean-Congo hemorrhagic fever, climate change, Kazakhstan, tick-borne diseases, One Health

## Abstract

*Background:* Crimean-Congo Hemorrhagic Fever (CCHF) is a severe zoonotic viral disease that poses significant public health challenges in Kazakhstan, particularly in the southern regions where it has been endemic for decades. The natural focus of CCHF in this region has expanded significantly, a trend likely exacerbated by climate change.

*Objective(s):* To analyze the outbreak trends of CCHF in four regions of Kazakhstan and assess the potential impact of climate variables on disease incidence.

*Methods:* This ecological study examined CCHF morbidity and mortality data from 2014 to 2023 in the Zhambyl, Turkistan, and Kyzylorda regions, and Shymkent City. Data were sourced from the national central data repository. Meteorological data (temperature and precipitation) were obtained from the Climatic Research Unit TS dataset. Regression analysis was performed to test the dependence of CCHF incidence on climate variables.

*Findings:* A total of 214 CCHF cases were reported in the study regions over the decade, with an overall case fatality rate of 21.5%. In the Kyzylorda region, a strong positive correlation was observed between climate indicators and annual case numbers (R-squared = 0.8969, *P* = 0.011). However, in Zhambyl and Turkistan regions, no significant associations emerged between climate variables and incidence during this period. Both average and extreme temperatures showed a gradual increase across all regions over the 10-year period.

*Conclusions:* While the direct correlation between climate variables and CCHF incidence varied by region, long-term temperature trends likely influence tick ecology. The findings highlight the complexity of evaluating CCHF dynamics solely through a climate lens and underscore the need for a One Health approach, improved surveillance, and cross-border cooperation to mitigate this growing public health threat.

## Introduction

Climate change is increasingly recognized as a serious threat to global health, including the spread of infectious diseases. Rising temperatures and changing precipitation patterns can extend the seasonal transmission window of vector-borne diseases and shift the geographic area of vectors and hosts [1]. For example, warmer winters and earlier springs have been shown to lengthen the active period of arthropod vectors, effectively prolonging transmission seasons [1]. Concurrently, climate anomalies can influence human behavior (such as increased outdoor exposure or agricultural practices), which in turn increases contact with disease vectors [2, 3]. Climate change is not just an environmental crisis but also a rapidly escalating public health emergency, especially in vulnerable regions. Central Asia, for instance, is highly susceptible to climatic shifts due to its dependence on glacier- and snowmelt-fed river basins. Recent high-resolution climate projections indicate the region could experience warming exceeding 4°C by the late 21st century, with drastic implications for water availability in the Amu Darya and Syr Darya river basins [4]. Dynamical downscaling studies show that under high-emission scenarios, the frequency of extreme precipitation events in Central Asia is projected to surge—the number of days with >20 mm rainfall may increase by over 90 days by century’s end [4]. These changes portend more frequent flooding and drought cycles, posing direct threats to food security, infrastructure, and public health in the region. Ultimately, climate change in Central Asia is expected to intensify environmental stresses and increase the risk of climate-sensitive diseases.

Within this context of profound environmental change, the epidemiology of zoonotic diseases such as Crimean-Congo Hemorrhagic Fever (CCHF) is drawing growing concern. CCHF, caused by an Orthonairovirus (family Nairoviridae), is primarily transmitted to humans through bites from infected *Hyalomma* ticks or contact with the blood and tissues of infected livestock [5]. Clinically, the disease begins suddenly with fever and muscle pain, and may progress to hemorrhagic symptoms. Case fatality rates vary by setting but usually range from 10% to 40% [6].

As climate patterns become less predictable, the distribution and seasonality of vector-borne diseases are shifting [1]. CCHF illustrates this well: rising temperatures and altered rainfall patterns allow *Hyalomma* ticks to survive in new areas, remain active longer, and potentially transmit the virus more efficiently [7, 8]. Warmer and drier conditions favor tick reproduction, while sufficient ground moisture remains essential for their survival, since these ticks desiccate easily [8]. Periods of heat coupled with reduced rainfall have been linked to expanding tick populations and prolonged CCHF seasons [7].

Field observations in endemic regions show that particularly hot years often bring more cases, as higher temperatures accelerate tick development and increase contact with humans [7]. Extreme climate events, particularly droughts, compromise the health and resilience of both livestock and human populations by causing malnutrition, destroying habitats, and forcing displacements, which collectively increase susceptibility to infection [2, 3]. Thus, climate influences CCHF risk in multiple ways: directly by shaping tick ecology and indirectly by affecting host susceptibility.

CCHF is endemic across Africa, southern and eastern Europe, the Middle East, and Asia [8, 9]. More than 30 countries have reported human cases, and the disease continues to spread into new areas. Spain, for instance, confirmed its first locally acquired cases in 2016, marking the virus’s arrival in temperate Europe [8]. Globally, 3,610 cases of CCHF were documented in 2023, resulting in a mortality rate of 8.1%. Asia represented the majority of these cases, accounting for 98.7%, with Kazakhstan alone reporting 40 cases. Kazakhstan’s lethality rate for CCHF stood at 17.5%, which is considerably higher than the worldwide average [10]. Central Asia is one of the world’s largest and most significant natural foci of CCHF, accounting for a substantial proportion of the global disease burden [11]. A comprehensive analysis of cases from 1944 to 2021 found that of 2,313 cases reported across Central, Eastern, and Southeastern Asia, 2,026 (88%) occurred in Central Asian countries. In Kazakhstan, CCHF has been recognized since 1948, with long-standing endemic foci in the southern regions of Kyzylorda, Turkistan, and Zhambyl, as well as the city of Shymkent [9, 12]. These arid and semi-arid landscapes are well suited to *Hyalomma* ticks. Between 1948 and 2013, 704 cases were reported nationwide [11]. In recent years, 10–20 sporadic cases have occurred annually, with a case fatality of about 14–15% [12].

Ticks such as *Hyalomma asiaticum, H. scupense,* and *H. anatolicum* dominate in Kazakhstan’s desert-steppe environments, feeding on small mammals during their immature stages and on livestock as adults [12]. This ecology ties the disease closely to pastoral livelihoods. Farmers, herders, and veterinarians face higher infection risks due to regular exposure to ticks or animal blood [7, 9]. Many human cases arise from tick bites during husbandry or from handling carcasses during home slaughter of sheep or cattle. The persistence of these foci over decades reflects the combination of favorable climate, abundant hosts, and close interaction between people, animals, and ticks.

**Figure 1:**
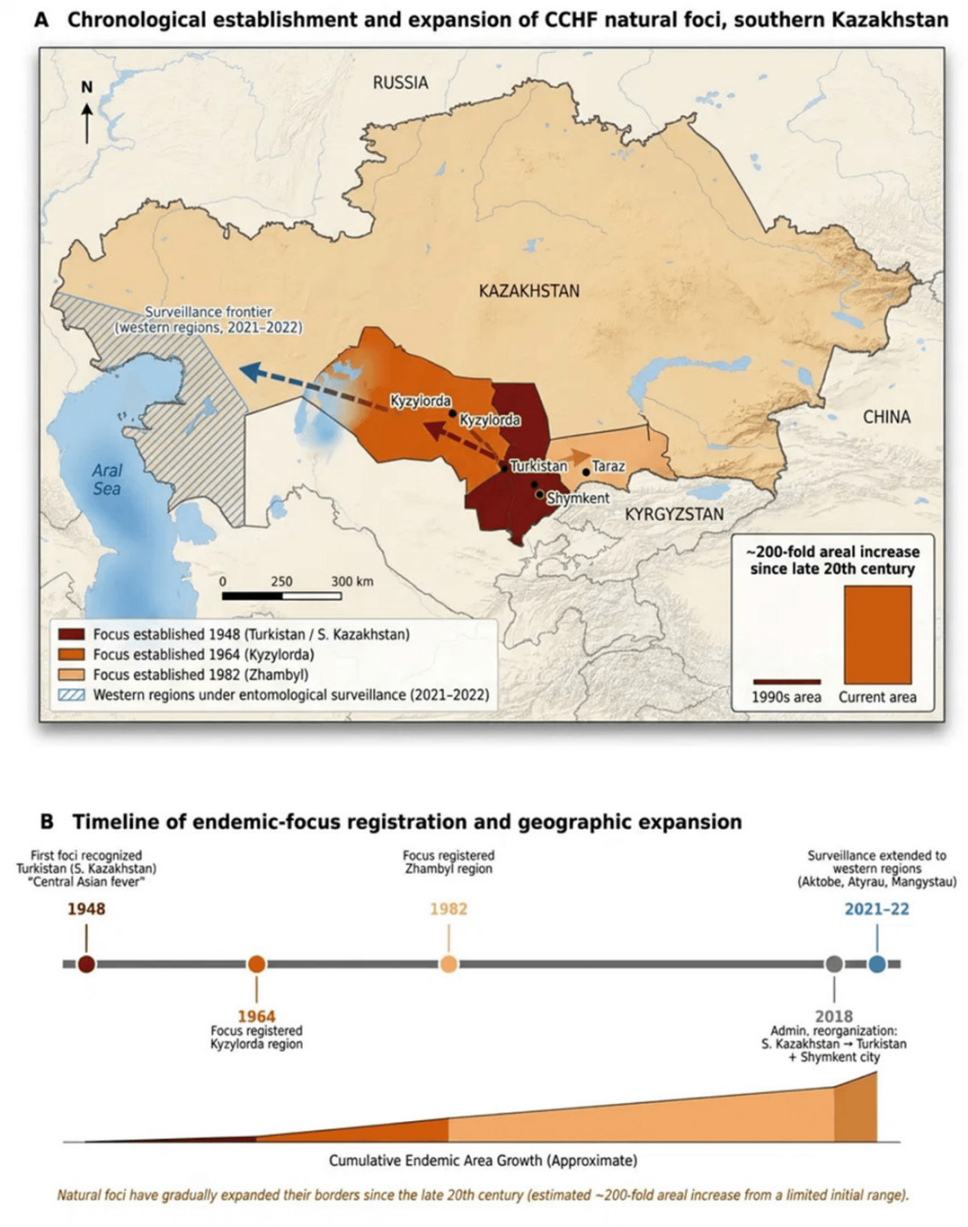
Chronological establishment and expansion of CCHF natural foci in Southern Kazakhstan 1948–2022.

Recent studies suggest that climate change is further expanding these endemic areas. Recent analysis indicates that the geographic extent of CCHF foci in southern Kazakhstan has increased approximately 200-fold since the late 20th century, driven largely by climate-induced changes in *Hyalomma* tick ecology, though this expansion occurred from a limited initial range [9, 12] (Figure 1).

While the exact figure may be uncertain, there is consensus that the area of CCHF vectors in Central Asia has increased substantially in recent years [12]. Modeling studies suggest that *H. marginatum* ticks struggle to survive in regions where the warm-season water vapor deficit is above ~15 hPa. In contrast, more humid environments provide favorable conditions for these ticks to persist [8]. With ongoing climate change, parts of Central Asia that were once too dry may now become sufficiently humid during spring and fall to support tick populations. At the same time, milder winters are reducing tick mortality, allowing more of them to survive through the cold season [8].

In a changing climate, previously inhospitable areas of Central Asia may become humid enough in spring or fall to support *Hyalomma* ticks, while milder winters reduce tick mortality and allow more ticks to overwinter successfully [8]. These climate-driven shifts are already apparent in CCHF’s spread beyond its traditional endemic zones in Africa, the Middle East, and South-West Asia. Notably, new CCHF outbreaks have been recorded in the past two decades in regions with no prior history of the disease, underscoring the virus’s widening range. The appearance of CCHF in Spain and elsewhere in Western Europe exemplifies this trend [8].

Without effective adaptation measures, countries such as Kazakhstan, Uzbekistan, and Tajikistan may face rising case numbers and further expansion of endemic zones. Addressing this evolving risk requires stronger surveillance, more interdisciplinary research, and proactive public health measures. We hypothesized that climate variables, particularly temperature and precipitation patterns, significantly influence CCHF incidence in endemic regions of Kazakhstan. To test this hypothesis, we analyzed outbreak trends of CCHF across four southern regions of Kazakhstan and assessed the relationship between meteorological variables and annual disease incidence over a 10-year period (2014–2023).

## Methods

### Research design

This was an ecological study based on available CCHF incidence data and meteorological variables.

### Study population and area

The study was conducted in the Republic of Kazakhstan, the world’s largest landlocked country, situated in Central Asia. Within Kazakhstan, CCHF is primarily endemic to the southern territories, specifically the Zhambyl, Turkistan, and Kyzylorda regions, as well as the city of Shymkent. Geographically, these regions form a contiguous area in the south of the country, bordering Kyrgyzstan and Uzbekistan. The Zhambyl region is located in the southeast, Turkistan and the megacity Shymkent are in the south-central part, and the Kyzylorda region lies to the southwest.

These southern regions present ideal ecological conditions for the proliferation of *Hyalomma* ticks, the principal vectors for the CCHF virus. The climate is sharply continental and ranges from semi-arid to arid, characterized by significant temperature fluctuations, with long, hot, and dry summers (+35 to +45°C) and cold winters. This climate pattern is highly conducive to the *Hyalomma* tick’s life cycle, as high ambient temperatures are necessary for egg development and questing activity. Furthermore, the landscape is dominated by deserts, semi-deserts, and vast steppe ecosystems. This environment supports not only the ticks themselves but also a wide range of hosts required for their survival across different life stages. Immature ticks primarily feed on small mammals like hares and birds, while adult ticks parasitize large domestic animals. The prevalence of traditional livestock husbandry ensures a stable and abundant source of hosts for adult *Hyalomma* ticks, thereby sustaining the enzootic cycle of CCHF.

According to recent demographic data, the estimated population for Kyzylorda Region is 841,500; for Zhambyl Region 1,221,400; and the combined population of Turkistan Region and Shymkent City is approximately 3,331,900. This brings the total population in the high-risk study area to over 5.3 million people.

### Study period

A 10-year study period (2014–2023) was selected to capture recent climate variability while retaining adequate temporal resolution for identifying climate-driven trends in CCHF epidemiology. The choice is further motivated by the accelerating pace of warming in the region. Kazakhstan has warmed substantially faster than the global mean, with mean annual surface air temperature rising by approximately 0.31°C per decade over 1950–2020 across 110 meteorological stations [12]. National monitoring indicates a rate of roughly 0.4°C per decade since the mid-1970s, with warming becoming particularly pronounced after 2010 [13]. While longer periods may capture additional climate cycles, the selected decade provides a robust window for evaluating contemporary climate-disease associations in the context of ongoing global warming, consistent with similar recent eco-epidemiological studies in the region.

### Outcome data

For this review, we gathered data on CCHF morbidity from the central data repository of the Scientific and Practical Center for Sanitary and Epidemiological Expertise and Monitoring and the National Center for Public Health of the Ministry of Healthcare of the Republic of Kazakhstan. We examined morbidity and mortality data from 2014 to 2023. This information was provided as aggregated data, without any personal identity. As the research team did not have access to patient identities, this study was exempt from full ethical review under national guidelines, and individual informed consent was not required (see Ethics and Consent statement below).

The dependent outcome variable in this study was the confirmed cases of CCHF, in Zhambyl, Kyzylorda, South Kazakhstan regions from 2014 to 2018, and Zhambyl, Kyzylorda, Turkistan regions and Shymkent city in 2019–2023, due to the renaming of the South Kazakhstan region to Turkistan and the designation of the city of Shymkent as a separate geographic zone. The number of cases diagnosed yearly was used in statistical models.

### Meteorological data

To analyze the climatic context of the study area, key meteorological data were sourced from the World Bank Group’s Climate Change Knowledge Portal (CCKP). For this study, historical observational data were utilized, specifically from the Climatic Research Unit (CRU) TS dataset. The CRU TS dataset offers a high-resolution gridded time series of monthly climate data for all land areas globally. From this dataset, the following synoptic variables were obtained for the period of analysis: Mean, Minimum, and Maximum Daily Temperature (°C), and Annual Precipitation (mm).

### Statistical analysis

To perform regression analysis to test the dependence of CCHF incidence on climate variables, we used R software.

## Results

Overall, 214 cases of CCHF were reported in Kazakhstan during the study period: 46 in Zhambyl region, 76 in Kyzylorda region, and 92 in South Kazakhstan/Turkistan regions and Shymkent city. The case fatality rate of CCHF in this study is 21.5%.

In the Zhambyl region, the annual average mean surface temperature ranged from 9.6°C in 2014 to 12.07°C in 2023 (Table 1). The temperature generally increased over the years, with a noticeable rise in 2023. The average minimum surface air temperature ranged from 3.37°C in 2014 to 5.93°C in 2023. Average maximum surface air temperature ranged from 15.86°C in 2014 to 18.24°C in 2023. Annual precipitation fluctuated significantly, ranging from 191.41 mm in 2021 to 320.49 mm in 2016. The CCHF incidence rate varied over the years, with a sharp increase in 2023 (12 cases). Both average mean and extreme temperatures showed a gradual increase over the 10-year period. As detailed in Table 4, the regression model for Zhambyl region had low explanatory power (R-squared = 0.3531) and was not statistically significant (*P*-value = 0.6337), indicating that CCHF incidence in this region was not significantly related to the analyzed climate data.

**Table 1:** Zhambyl region CCHF incidence and climate data for 2014–2023.

VARIABLE	2014	2015	2016	2017	2018	2019	2020	2021	2022	2023
Annual average mean surface temp (°C)	9.6	11.6	11.31	10.64	10.28	11.29	10.51	11.25	11.61	12.07
Average min surface air temp (°C)	3.37	5.26	5.24	4.51	4.13	5.19	4.41	5.02	5.71	5.93
Average max surface air temp (°C)	15.86	17.1	17.42	16.8	16.45	17.42	16.64	17.51	17.54	18.24
Annual precipitation (mm)	239.52	265.17	320.49	257.72	238.61	205.6	217.74	191.41	263.27	227.63
CCHF incidence rate (cases)	1	5	7	6	9	1	2	1	2	12

For the Kyzylorda region, the annual average mean surface temperature ranged from 9.88°C in 2014 to 12.77°C in 2023 (Table 2). The average minimum surface air temperature ranged from 3.39°C in 2014 to 6.29°C in 2023. The average maximum surface air temperature ranged from 16.38°C in 2014 to 19.27°C in 2023. Annual precipitation ranged from 96.69 mm in 2021 to 195.66 mm in 2016. The CCHF incidence rate varied, with a sharp increase in 2022 (20 cases) and a slight decrease to 13 cases in 2023. The regression model for Kyzylorda region had high explanatory power (R-squared = 0.8969) and was statistically significant (*P*-value = 0.01108) (Table 4). This suggests that CCHF incidence in the Kyzylorda region is strongly related to climate data, particularly temperature and precipitation. The increase in CCHF cases in 2022 coincides with higher temperatures and moderate precipitation levels.

**Table 2:** Kyzylorda region CCHF incidence and climate data for 2014–2023.

VARIABLE	2014	2015	2016	2017	2018	2019	2020	2021	2022	2023
Annual average mean surface temp (°C)	9.88	11.48	11.76	11.06	11.31	11.62	11.34	11.72	11.82	12.77
Average min surface air temp (°C)	3.39	5.47	5.69	4.79	3.98	5.25	4.97	5.3	5.78	6.29
Average max surface air temp (°C)	16.38	17.52	17.85	17.35	16.66	18.02	17.74	18.16	17.87	19.27
Annual precipitation (mm)	123.38	195.66	140.2	122.07	133.18	111.99	114.32	96.69	128.46	128.43
CCHF incidence rate (cases)	1	1	5	2	4	9	9	12	20	13

For the South Kazakhstan (Turkistan and Shymkent city) region, the annual average mean surface temperature ranged from 11.99°C in 2014 to 14.48°C in 2023 (Table 3). The average minimum surface air temperature ranged from 5.39°C in 2014 to 8.11°C in 2023. The average maximum surface air temperature ranged from 18.62°C in 2014 to 20.89°C in 2023. Annual precipitation ranged from 236.04 mm in 2021 to 365.54 mm in 2016. The CCHF incidence rate varied, with a sharp increase in 2022 (19 cases). The regression model had high explanatory power (R-squared = 0.789) but was not statistically significant (*P*-value = 0.05) (Table 4). This suggests that CCHF incidence in the South Kazakhstan region is not significantly related to the analyzed climate data.

**Table 3:** South Kazakhstan (Turkistan and Shymkent city) region CCHF incidence and climate data for 2014–2023.

VARIABLE	2014	2015	2016	2017	2018	2019	2020	2021	2022	2023
Annual average mean surface temp (°C)	11.99	13.35	13.67	12.9	12.82	13.68	12.7	13.66	13.9	14.48
Average min surface air temp (°C)	5.39	7.14	7.27	6.37	6.37	7.34	6.37	7.23	7.93	8.11
Average max surface air temp (°C)	18.62	19.6	20.11	19.46	19.3	20.07	19.05	20.13	19.9	20.89
Annual precipitation (mm)	311.24	341.43	365.54	302.76	265.99	270.02	271.17	236.04	342.95	261.52
CCHF incidence rate (cases)	4	6	15	8	5	9	6	6	19	14

**Table 4:** Statistical analysis of CCHF incidence and climate variables (2014–2023).

METRIC	KYZYLORDA	ZHAMBYL	SOUTH KAZAKHSTAN
**Climate Influence**	Strong	Weak	Moderate
**Statistical Significance**	Yes (*P* = 0.011)	No (*P* = 0.634)	Borderline (*P* = 0.050)
**Predictive Capacity**	High (R² = 0.90)	Low (R² = 0.35)	Moderate (R² = 0.79)
**Total Cases**	76	46	92
**Peak Year**	2022 (20 cases)	2023 (12 cases)	2022 (19 cases)

## Discussion

Our findings highlight the central role of climate in shaping the ecology of CCHF in southern Kazakhstan. Temperature is a key driver: *Hyalomma* ticks need sufficient warmth to develop and actively seek hosts. Field studies show that *H. marginatum* adults become active in spring once mean temperatures reach about 10–12°C, with host-seeking peaking between 22°C and 27°C under humid conditions [14]. Warmer conditions speed up their life cycle, allowing more frequent blood meals and reproduction within a season. However, excessive heat can suppress activity. Thus, both heat and moisture are essential for ticks to thrive, and climate change alters this balance in different ways across local landscapes.

Many studies have reported that warming and shifting rainfall patterns drive the expansion of *Hyalomma* ticks and CCHF cases in endemic areas [7]. Our analysis, however, found mixed results in Kazakhstan. In Kyzylorda region, we observed a strong positive correlation between climate indicators and annual case numbers, consistent with earlier research. In Zhambyl and Turkistan, by contrast, no significant associations emerged between climate variables and incidence over 2014–2023. This apparent contradiction may reflect the small number of cases in Kazakhstan, which limits statistical power and allows random fluctuations or surveillance differences to mask climate signals. Shifts in diagnostic practices or awareness campaigns could also influence reported numbers independently of climate. These findings do not rule out climate effects; rather, they highlight the difficulty of detecting them in short-term datasets and the need for stronger surveillance.

While our 10-year study period provides valuable insights into recent climate-driven changes in CCHF distribution, we acknowledge that this timeframe may not capture longer-term multi-decadal climate oscillations that operate on 20–30-year timescales. Future studies incorporating longer temporal datasets would strengthen our understanding of climate-driven trends in CCHF epidemiology. Nevertheless, the temporal progression of endemic zones within Kazakhstan expanding approximately 200-fold since the late 20th century (Figure 1) strongly indicates a changing ecological baseline conducive to tick proliferation.

Patterns in Kazakhstan resemble those in neighboring countries but also show important distinctions. Kazakhstan’s endemic zones border similar foci in Uzbekistan, where sporadic outbreaks have been reported [15]. Western Uzbekistan, despite its arid climate, supports *Hyalomma* ticks around irrigated farmlands and villages. The same vector species are common across both countries. In Kyrgyzstan, high-altitude zones have cooler summers and harsh winters unfavorable to *Hyalomma* ticks, but the lowland Ferghana Valley and southern foothills provide suitable conditions and have seen periodic CCHF cases [16, 17]. Across the region, pastoral communities remain at highest risk. Differences in reported incidence likely stem less from ecology than from surveillance intensity [15]. The shared ecology across borders reinforces the point that CCHF is a regional problem that requires collective responses.

Across the broader region, one important insight is that strong surveillance and reporting systems are critical for understanding climate–disease relationships. The absence of a mandatory livestock infection reporting system in Kazakhstan is recognized as a key weakness that could hinder timely detection of CCHF outbreaks. If farmers and animal owners are not legally required to report tick infestations or sudden livestock illnesses, veterinary authorities may be “blind” to important early signals. Limited and delayed data also impair our ability to perform rigorous statistical analyses: under-reporting can dilute true correlations between climate variables and incidence. Research from other CCHF-endemic regions often reports stronger associations between warming trends and tick-borne disease incidence [7]. One hypothesis for why Kazakhstan’s data did not fully align is that baseline climate and host factors differ. More granular data would help unravel these nuances.

From a public health perspective, our study reinforces the need for cross-border cooperation and a One Health approach to CCHF control in Central Asia. No single country can effectively mitigate CCHF risks in isolation, given the regional movement of hosts and vectors. Joint surveillance and response strategies are essential. Harmonizing policies on livestock movement is another key area. International agencies like the World Health Organization and the World Organization for Animal Health can facilitate these collaborations. Adopting a One Health perspective—integrating human, animal, and environmental health—is crucial because CCHF maintenance involves all three domains. An interdisciplinary surveillance network that tracks human cases, tests livestock for antibodies, and samples ticks in the environment would provide a much more comprehensive early warning system than siloed efforts.

Environmental monitoring also fits into the One Health framework, since these factors influence where CCHF might flare up. By pooling data from all these sources into a single analytic platform, “hotspots” of elevated risk can be identified and targeted for intensified control. This holistic strategy addresses what is often the “silent” portion of the CCHF cycle—virus circulation in ticks and animals that goes unnoticed until humans fall ill. By intervening earlier in that cycle, the spillover to humans could be reduced.

Another practical implication of our work is the opportunity to develop early warning systems for CCHF outbreaks by leveraging climate and remote-sensing data. Since certain meteorological patterns can predict a forthcoming surge in tick activity, these indicators could be used to alert public health authorities weeks or months in advance. Health agencies can incorporate real-time weather surveillance and satellite-derived indices into a predictive model. One study in Turkey successfully used temperature and humidity trends to forecast the start and peak of the CCHF season [18]. In Central Asia, we envision a similar alert system where meteorological services, epidemiologists, and ecologists collaborate to generate risk maps each season [19, 20]. An effective early warning would enable preemptive actions: stockpiling medical supplies, intensifying acaricide spraying, and issuing public advisories.

Finally, effective risk communication and community engagement are cornerstones of CCHF prevention in the face of both climate and non-climate drivers. Public health authorities in Central Asia should collaborate with agricultural agencies and local leaders to disseminate clear, practical guidance each year before the CCHF season. Key messages include how to avoid tick bites, proper tick removal, and safe animal slaughtering practices. Many CCHF cases in endemic villages occur due to delayed health-seeking. Thus, educating communities about early symptom recognition and urgent care-seeking is vital. We also note the importance of training healthcare workers to recognize CCHF and use strict infection control when treating suspected patients.

## Study Limitations

This study has several limitations. First, there are likely unmeasured confounders such as wild host population dynamics. Second, we did not have high-resolution vector data and inferred tick activity from climate proxies [7, 19]. Without directly observing ticks and testing them for the virus, we rely on climate surrogates, which introduce uncertainty. Likewise, our measure of host exposure was coarse. This kind of spatial averaging can lead to ecological fallacy or misclassification. Third, there is potential bias in case detection and reporting. Milder cases or subclinical infections likely go undiagnosed. If less severe cases are systematically missed, then our analysis skews toward the more severe end of the clinical spectrum. Lastly, while we focused on several meteorological variables, other environmental changes and socioeconomic trends could be contributing to CCHF incidence but were outside of our scope. Our ecological study design is useful for generating hypotheses on broad associations. Despite these limitations, our study contributes valuable insight into CCHF in Central Asia’s context and raises new questions.

## Conclusion

Despite this study finding no direct correlation between climate variables and CCHF incidence in all regions, considerable evidence from both Kazakhstan and international research suggests that long-term temperature trends may still influence tick ecology. The apparent contradiction may highlight underlying governance and infrastructure challenges rather than definitively ruling out climate effects. Moving forward, a One Health-based, multi-sectoral strategy could be crucial for improving disease monitoring and policy development. Key recommendations include implementing mandatory livestock surveillance, enhancing data integration through centralized platforms, refining climate metrics to capture daily extremes and vegetation indices, and extending monitoring periods to detect cumulative climate effects. Taken together, these findings highlight the complexity of evaluating CCHF dynamics solely through a climate change lens. Institutional deficiencies, particularly gaps in surveillance, can obscure or distort environmental signals. Nevertheless, the burden of CCHF in Kazakhstan remains substantial, with continued reports of virus range expansion and sustained exposure risks for high-risk occupational groups. Strengthening Kazakhstan’s epidemiological infrastructure, improving climate-informed disease modeling, and adopting a unified One Health approach will be key to mitigating the public health challenges posed by CCHF.

## Data Availability

“The CCHF morbidity and mortality data analyzed in this study are held by the Scientific and Practical Center for Sanitary and Epidemiological Expertise and Monitoring and the National Center for Public Health of the Ministry of Healthcare of the Republic of Kazakhstan and are available from the corresponding author upon reasonable request, subject to institutional approval. Meteorological data are publicly available from the World Bank Group’s Climate Change Knowledge Portal (https://climateknowledgeportal.worldbank.org).”

## Data Access Verification

All authors had access to the data and a role in writing the manuscript.
